# Nonlinear Quantitative Radiation Sensitivity Prediction Model Based on NCI-60 Cancer Cell Lines

**DOI:** 10.1155/2014/903602

**Published:** 2014-06-17

**Authors:** Chunying Zhang, Luc Girard, Amit Das, Sun Chen, Guangqiang Zheng, Kai Song

**Affiliations:** ^1^School of Chemical Engineering and Technology, Tianjin University, Tianjin 300072, China; ^2^Hamon Center for Therapeutic Oncology, University of Texas Southwestern Medical Center, Dallas, TX 75390, USA; ^3^Department of Pharmacology, University of Texas Southwestern Medical Center, Dallas, TX 75390, USA; ^4^Department of Radiation Oncology, University of Texas Southwestern Medical Center, Dallas, TX 75390, USA; ^5^Department of Clinical Sciences, University of Texas Southwestern Medical Center, Dallas, TX 75390, USA

## Abstract

We proposed a nonlinear model to perform a novel quantitative radiation sensitivity prediction. We used the NCI-60 panel, which consists of nine different cancer types, as the platform to train our model. Important radiation therapy (RT) related genes were selected by significance analysis of microarrays (SAM). Orthogonal latent variables (LVs) were then extracted by the partial least squares (PLS) method as the new compressive input variables. Finally, support vector machine (SVM) regression model was trained with these LVs to predict the SF2 (the surviving fraction of cells after a radiation dose of 2 Gy *γ*-ray) values of the cell lines. Comparison with the published results showed significant improvement of the new method in various ways: (a) reducing the root mean square error (RMSE) of the radiation sensitivity prediction model from 0.20 to 0.011; and (b) improving prediction accuracy from 62% to 91%. To test the predictive performance of the gene signature, three different types of cancer patient datasets were used. Survival analysis across these different types of cancer patients strongly confirmed the clinical potential utility of the signature genes as a general prognosis platform. The gene regulatory network analysis identified six hub genes that are involved in canonical cancer pathways.

## 1. Introduction

Radiation therapy (RT) is one of the important treatment modalities used in cancer treatment either alone or in conjunction with chemotherapy and surgery. Approximately 50% of all cancer patients receive RT during their course of treatment [1]. It has been demonstrated that radiation can suppress tumor growth either by inhibiting cell proliferation or by inducing cell death or both [2]. However, the radiation response of different tumors varies greatly. This is true even for the same type of tumor in different patients [3]. Thus, personalized treatment necessitates quantitative and accurate prediction of tumor cell response to RT.

The advent of microarray technology has had a significant effect on oncology research. Studies over recent decades attempted to correlate the relationship between cellular radiation sensitivity and expression levels of some important genes [4]. Most published studies identified radiation sensitivity-related genes from a single homogenous cancer tumor type, for example, uterine cervical cancer [5], oral squamous cell carcinoma [6], colorectal cancer [7], and lung cancer [8]. It is generally accepted, however, that the gene signature will be more robust and general if derived from heterogeneous cell lines involving multiple tissues of origin. The most comprehensive studies used the NCI-60 panel of cell lines.

The NCI-60 panel is the most extensively characterized set of cancer cell lines and a public resource that is widely used as a platform for drug discovery [9]. Some NCI-60 platform based investigations demonstrated that tumor response to RT was predictable by means of gene expression profiling. Using NCI-60 cancer cell lines, Amundson et al. [10] performed large-scale comparisons of gene expression variations in response to different doses (2, 5, and 8 Gy) of *γ*-ray radiation. Kim et al.[11] integrated four published microarray studies on NCI-60 cell lines in an effort to identify common radiation sensitivity-related gene signature across all available microarray platforms. Torres-Roca et al. [12] used significance analysis of microarrays (SAM) [13] to select genes that were significantly correlated with radiation sensitivity measured as a surviving fraction at 2 Gy (SF2). They then used the selected genes to construct a linear prediction model of SF2. The leave-one-out (LOO) method was applied on 35 cell lines. This resulted in SF2 prediction of 22 cell lines with <10% errors. Based on this study [12], Eschrich et al. [14] used a least squares fit algorithm to construct a linear model to predict SF2s with gene expression values and other biological parameters. They selected 500 genes from 7,168 probe sets. Then by using GeneGo and MetaCore, a scale-free network of 500 genes was created highlighting several important biological pathways. Finally, they provided a linear equation between SF2s and expression values of ten network hub genes.

These previous studies [5–8, 10–12, 14] made a sincere effort in understanding association of gene expression and radiation response with the ultimate goal to personalize radiation treatment of different cancers. Improvement in quantitative prediction of* in vitro* radiation sensitivity by linear regression model achieved by Torres-Roca et al. [12] and Eschrich et al. [14] divided cell lines into sensitive or resistant groups according to their predicted SF2 values. Such linear regression models are obviously not sufficiently enough for the detection of multiple and combinational nonlinear gene interactions [15]. In our study, therefore, we aim at further improving quantitative and accurate prediction of radiation response represented by SF2 values. The precise survival prediction of patients, then, can be achieved by using the predicted SF2 and other clinical parameters, that is, age, tumor stage, and so forth. Within NCI-60 platform there are cell lines of nine different types of cancer and we chose this platform to build our prediction model.

It is known that high dimension and multicollinear relationships among genes, inherent in microarray data, can reduce the usefulness of widely used analysis methods [16]. As a first step, we therefore used SAM to select highly significant radiation response-related genes. Then, partial least squares (PLS) [17] algorithm was applied to extract latent variables (LVs). These two steps reduced the number of input variables (gene expression profiles) from 19,162 to 17 which effectively overcame the “dimensionality curse” problem. The orthogonality among LVs successfully removed the multicollinearity inherent in original gene expression values. Finally, a kernel-based regression model was generated for the SF2 prediction by using support vector machine (SVM) [18]. We obtained an accurate radiation sensitivity prediction model. The minimum prediction error was an exceptional “0”. The RMSE (root mean square error) was markedly reduced from 0.20 (of Torres-Roca et al. [12]) to 0.011. The prediction accuracy was improved from 62% to 91%. Survival analysis across three datasets demonstrated the robustness of our gene expression signature. This could have immense clinical potential. Additionally, the gene regulatory network analysis identified six hub genes which are involved in known cancer pathways.

## 2. Materials and Methods

### 2.1. NCI-60 Platform

In the late 1980s the US National Cancer Institute selected 60 (NCI-60) cell lines representing nine tumor types as an* in vitro* drug-discovery platform [9]. They contained breast, central nervous system, colon, leukemia, lung, melanoma, ovarian, prostate, and renal cancers. Continued screening of potential adjuvant agents and molecular characterization of these cell lines were informative and made them a valuable resource for research of drug reaction, tumor growth inhibition, and so forth [19]. Accordingly, in our study, we used it as the platform to assess the performance of our method.

Radiation response is defined as the survival fraction of tumor cells at a given dose of *γ*-ray radiation. SF2 represents the surviving fraction of cells after radiation dose of 2 Gy *γ*-rays and is widely used as measurement of radiation sensitivity* in vitro *due to reproducibility and the clinical relevance (in clinical practice, patients generally receive dose equivalent of 2 Gy of radiation per fraction). SF2 is a continuous value ranging from 0 to 1. The lower the SF2 value of a cell line is, the more sensitive it is to radiation. SF2 values of NCI-60 cell lines were obtained from previously published data [14].

Gene expression profiles of NCI-60 cell lines were obtained from gene expression omnibus (GEO) (series accession number GSE32474 [20]). The expression data of cell line BREAST_MDN were not available; the detailed information of the remaining 59 cell lines and corresponding measured SF2 values are shown in Supplementary Table S1 (the fourth column) (see Table S1 in Supplementary Material available online at http://dx.doi.org/10.1155/2014/903602).

### 2.2. Cancer Patient Datasets

To test the clinical significance of the genetic signature, we selected patients with glioma, colon cancer, and ovarian cancer. Supplementary Table S2 provides the summarized clinical parameters of these patients.

### 2.3. Procedure of Radiation Sensitivity Modeling

As a first step, SAM [13] was used for gene selection and dimension reduction. This method carries out gene specific *t*-test and computes a score of *d*_*j*_ for each gene *j*. The *d*_*j*_ score is used to measure the strength of the relationship between gene expression value and a response variable (radiation sensitivity). Genes with scores higher than the threshold are assumed to be significantly related to radiation sensitivity. A false discovery rate (FDR) [21] of 1% was used to control the proportion of genes incorrectly identified as significant.

In the second step, PLS [17] was used as a feature extraction technique to further reduce the input dimension. In PLS, orthogonal LVs are extracted by constructing linear combinations of the gene expression profiles. Therefore, the *n*-dimensional gene expression space is compressed into the *v*-dimensional LV-space, where *v* ≪ *n*. As a benefit of the orthogonality of LVs, the multicollinear relationship among genes was eliminated. At the same time, the interference of the noise was excluded.

**Figure 1:**
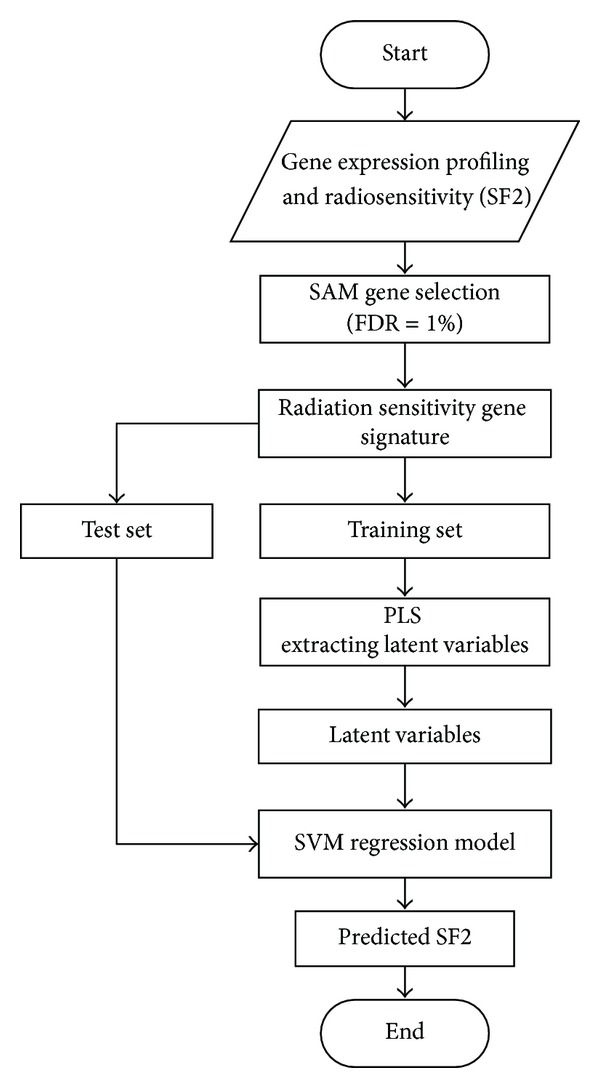
Flow chart of radiation sensitivity prediction model. Abbreviations: SF2—survival fraction at 2 Gy *γ*-ray radiation; SAM—significance analysis of microarrays; FDR—false discovery rate; PLS—partial least squares; and SVM—support vector machine.

Finally, the selected LVs were used as input variables to SVM regression algorithm [18, 22]. SVM maps the original input space into a higher dimensional *f* space using a nonlinear mapping. A linear model is then constructed in *f* space. As a result, the nonlinear relationship between the expression values of genes and the radiation response was modeled. The flow chart of our method is illustrated in Figure 1.

The details and parameter settings of SAM, PLS, and SVM are available in the Supplementary Material.

### 2.4. Microarray Data Preprocessing

Entrez gene identifiers (Entrez ID) from NCBI were used to match genes across different chip platforms. The chip probes that could not be matched with any HUGO [23] gene symbol were removed.

### 2.5. Measurement of SF2 Prediction Performance

The RMSE was used to evaluate the performance of the SF2 prediction model. It measures how close the predicted SF2s are to the measured SF2s. A lower RMSE indicates better prediction capacity of the regression model. The definition of RMSE is

(1) $$\begin{array}{c} \text{RMSE}=\sqrt{\frac{\sum_{i=1}^{m}{\left(\hat{Y_{i}}-Y_{i}\right)}^{2}}{m}}, \end{array}$$

where *Y*_*i*_ is the measured SF2 value of cell line *i*, $\hat{Y_{i}}$ is the corresponding predicted SF2, and *m* is the number of the cell lines (*m* = 59 in this study).

Ten rounds of 3-fold cross-validation were processed to train and test the prediction model. The final predicted SF2 values were determined by the average of these ten independent rounds.

### 2.6. Survival Analysis

Univariate Cox proportional hazard models were used to evaluate the association between patient survival risk and expression values of each of the signature genes. A patient's risk score was determined as the sum of all survival-related gene expression values multiplied by the corresponding univariate Cox regression coefficients. Patients were divided into high-risk and low-risk groups with the median of risk scores as the threshold value. Survival curves of high-risk and low-risk groups were estimated using Kaplan-Meier method and compared with log-rank tests [24].

The survival analysis for each type of cancer patient's dataset was conducted independently. All statistical tests were two-tailed. *P* values less than 0.05 were considered statistically significant.

## 3. Results and Discussion

### 3.1. Nonlinear SF2 Prediction Model Based on the NCI-60 Cell Lines

**Figure 2:**
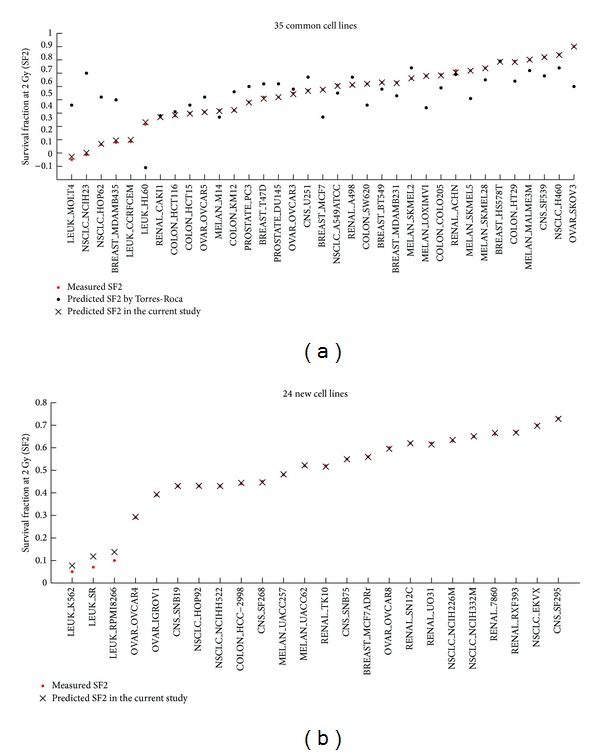
The measured and predicted SF2s of NCI-60 obtained by our method and by the model of Torres-Roca et al. Note: among 59 cancer cell lines used in the current study, only 35 were included in the study of Torres-Roca et al. (Figure 2(a)).

Quantitative prediction of RT response is of fundamental and practical interest in clinical research [4]. Here we used 19,162 genes as input variables but only 59 samples. This large imbalance among variables and samples may cause data-driven prediction methods to give unsatisfactory results. We used SAM analysis to select 129 genes correlated with radiation response (Supplementary Table S3). In order to overcome multicollinear relationship among genes, PLS was used to extract 17 orthogonal LVs as new input variables for the SVM regression model. The final predicted SF2 values are presented in Figure 2 and Supplementary Table S1 (the second column).

#### 3.1.1. Comparison with the Previous Studies

We compared our results with previously published results in the literature [12].

The minimum error between the predicted and observed SF2s of all 59 cell lines was an exceptional “0”, which corresponded to the calculated error for eight cell lines (BREAST_BT549, PROSTATE_DU145, BREAST_MCF7, CNS_SF539, COLON_SW620, CNS_SNB19, NSCLC_NCIHH522, and RENAL_SN12C). The maximum error of 0.048 was unique to the calculated error for the LEUK_SR cell line. The RMSE of the prediction model was only 0.011, which is much smaller than the RMSE of 0.20 as reported by Torres-Roca et al. [12]. RMSE is a measure of the differences between the predicted and observed SF2 values. Smaller RMSE values indicate smaller difference between observed and predicted values. The accuracy of our model was more than ten times that of the model provided by Torres-Roca et al. Figure 2 shows the observed and predicted SF2 values of NCI-60 cell lines calculated by our model and by Torres-Roca's model. According to our proposed model, differences between predicted and observed SF2 values were minimal in all 35 cell lines (Figure 2(a)). The only exception was the BREAST_HS578T cell line where the absolute prediction error obtained by our method was 0.005 compared with “0” obtained by Torres-Roca's model.

The work of Torres-Roca et al. presumed the predicted SF2s correct if they were within ±10% of the actual reported average SF2s or of their own measured SF2s. Accordingly, their model correctly predicted the SF2 values of 22 out of 35 cell lines resulting in an accuracy of 62%. In our study, using the same assumption, we achieved 91% accuracy and predicted SF2 values of 54 out of 59 cell lines correctly. This is a significant improvement of 29% over the Torres-Roca's published model.

Using the 17 LVs rather than 129 selected genes or the original 19,162 genes as the input variables to SVM can also speed up its regression process. The average time of training SVM using 17 LVs was 178 seconds which was much faster than the 534 seconds using 129 signature genes. All R codes of these programs were run on a PC with a 3.07 GHz Intel i7 CPU and 4 GB RAM.

#### 3.1.2. Comparison between SVM and Linear Regression Algorithm

**Figure 3:**
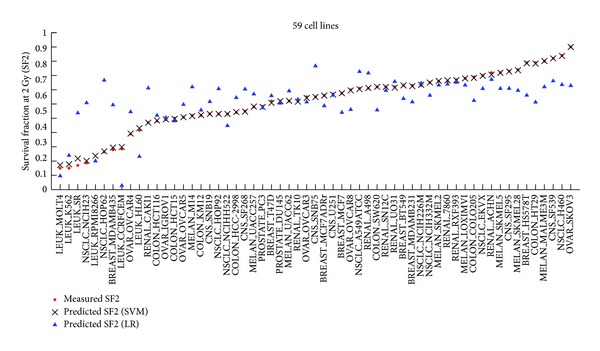
Comparisons between SVM regression and line regression (LR) algorithm.

In our proposed approach, SVM regression algorithm was used to develop the SF2 prediction model. The application of kernel function in SVM helps capture the nonlinear dependence relationship between gene expression and SF2s. To further verify this, we compared the results respectively derived from SVM and linear regression (LR) in Figure 3 and Supplementary Table S1 (the third column).

We found that the predicted SF2s by SVM were much closer to the measured SF2s than those of LR. Only in one cell line (LEUK_RPMI8266) was the absolute prediction error of LR (0) lower than that of SVM (0.037). However, the calculated RMSE of LR was up to 0.16 and the prediction accuracy was only 31%, which were clearly worse than the results of SVM (0.011 and 91%). Therefore, we conclude that the radiation sensitivity prediction model in the current study is especially suitable for exploring the nonlinear biological interactions.

### 3.2. Survival Analysis Based on the Gene Expression Signature

Since the NCI-60 panel has several different types of cancer-derived cell lines, we presume that the gene signature is applicable to all cancer types represented in the panel. We have selected glioma, colon, and ovarian cancer datasets for survival analysis. The gene expression signature was refined for each of them using the univariate Cox regression model. The survival analysis was then processed for the corresponding patients.

**Figure 4:**
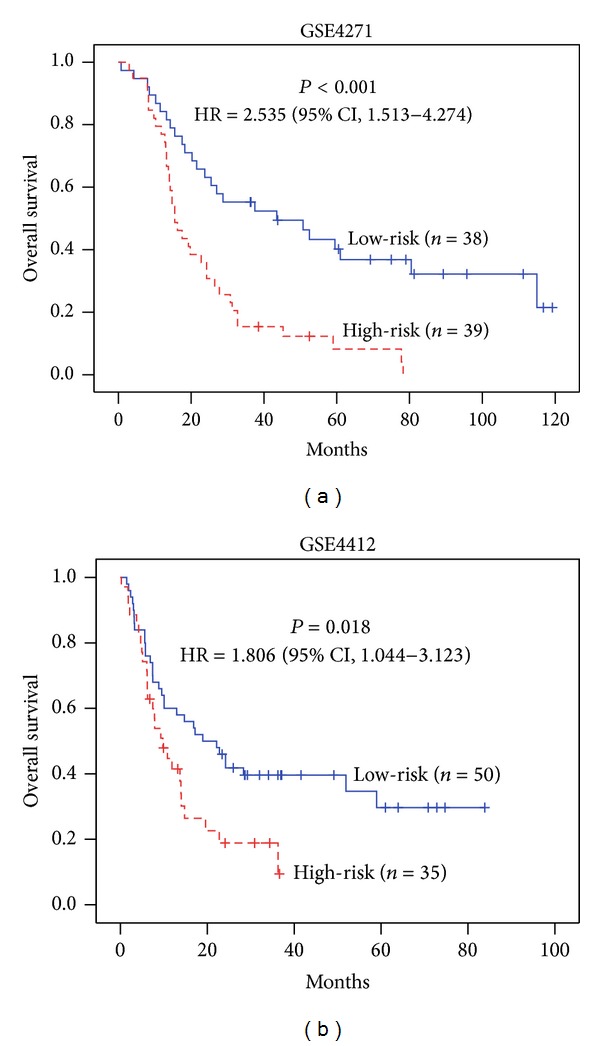
Survival curves of the glioma patients. Abbreviations: HR—hazard ratio; CI—confidence interval. The Cox regression model was trained with the 26 genes refined by the GSE4271 training dataset. The median of the estimated risk scores was used as the cutoff to divide the patients into high-risk and low-risk groups. *P* values were obtained from the log-rank test.

For glioma, dataset GSE4271 was used as the training set and dataset GSE4412 was used as the testing set. 26 out of 129 genes were selected as refined signature genes of glioma. They are summarized in Table 1. The survival analysis and KM curves of the patients with this 26-gene signature are displayed in Figure 4. It is apparent that high-risk group patients have significantly worse overall survival outcomes than low-risk group patients. The estimated hazard ratio (HR) between these two groups of GSE4271 is HR = 2.535 (*P* value < 0.001). The HR between these two groups of GSE4412 is HR = 1.806 (*P* value = 0.018).

The refined signatures for the colon and ovarian cancer are also shown in Table 1. The corresponding patient survival analysis results are shown in Supplementary Figures S1 and S2.

For colon cancer, KM curves show a significant difference in overall survival between the predicted high-risk and low-risk groups. In training set GSE17537, the HR between the two groups is 5.313 (*P* value = 0.001). In testing set GSE17536, the HR is 2.311 (*P* value = 0.001). For ovarian cancer, the refined 18-gene signature also predicts overall survival in training set GSE9891 (*P* value < 0.001) and testing set GSE17260 (*P* value = 0.020). The HR between the predicted high-risk and low-risk groups is 2.401 and 1.662, respectively. These results confirmed that the 129 genes actually covered the refined genetic signature of different types of cancers.

**Figure 5:**
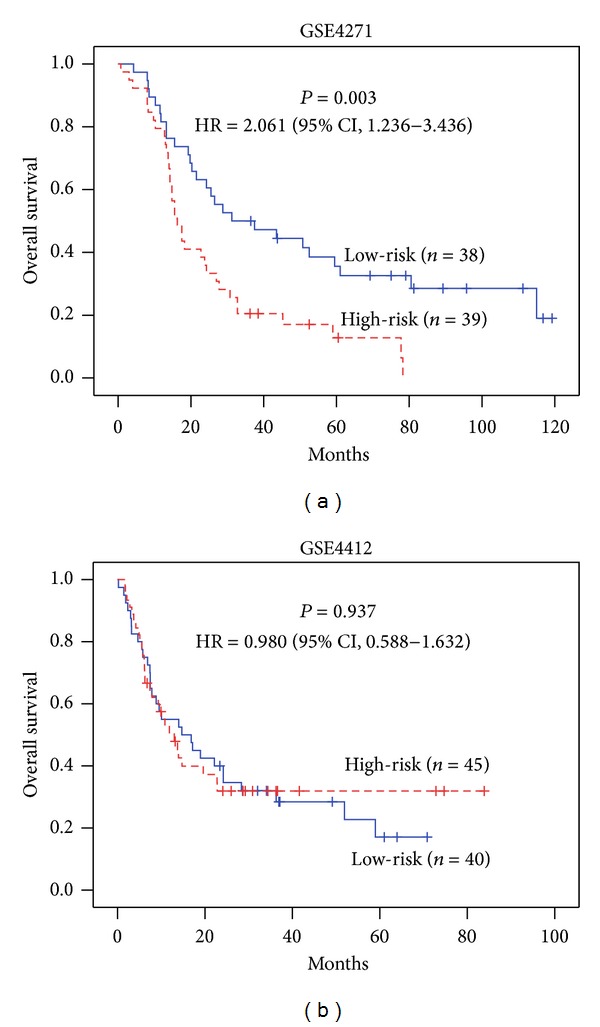
Survival analysis of the glioma patients using ten hub genes reported by Eschrich et al. Abbreviations: HR—hazard ratio; CI—confidence interval. The Cox regression model was trained by ten hub genes. The median of the estimated risk scores was used as the cutoff to divide the patients into high-risk and low-risk groups. *P* values were obtained from the log-rank test.

Eschrich et al. previously reported ten radiation-specific network hub genes using the same NCI-60 panel [14]. We performed survival analysis using these ten genes for the glioma, colon, and ovarian cancer patient datasets. The KM curves are shown in Figure 5, Supplementary Figures S3 and S4. The results show that the ten hub genes reported by Eschrich et al. only predict overall survival in two of the three types of cancer. The HR between predicted high-risk and low-risk groups in training datasets is shown below:

- glioma, 2.061 (*P* value = 0.003);
- colon cancer, 8.156 (*P* value < 0.001);
- ovarian cancer, 1.218 (*P* value = 0.291).

There is no significant difference between the predicted high-risk and low-risk groups in all testing datasets. The HR between predicted high-risk and low-risk groups of testing sets is shown below:

- glioma, 0.980 (*P* value = 0.937);
- colon cancer, 0.903 (*P* value = 0.664);
- ovarian cancer, 1.091 (*P* value = 0.699).

Although the survival analysis using the ten genes achieved outstanding results on the training sets, the results of the testing sets were unsatisfactory and suggested a limited predictive role of the ten hub genes in clinical applications.

### 3.3. Functional Network Analysis of the Selected Genes

**Figure 6:**
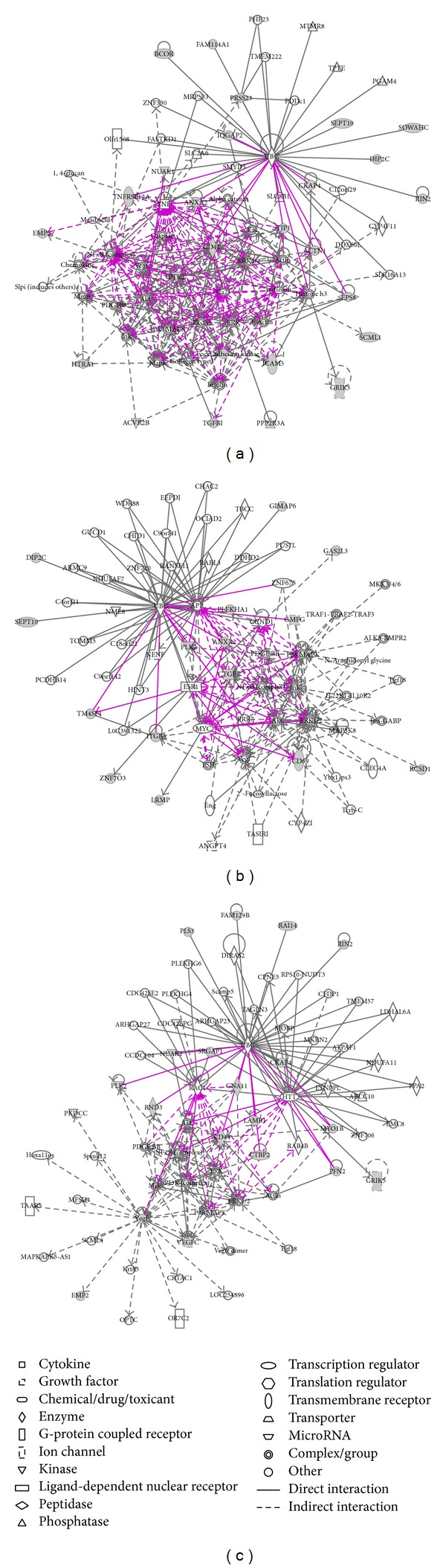
Gene regulatory networks of the survival-related genes generated by IPA. (a) 26-gene set for glioma; (b) 16-gene set for colon cancer; and (c) 18-gene set for ovarian cancer. Shapes represent different molecule types (details are illustrated in bottom middle panel). Filled shapes represent the survival-related candidate genes. Solid line indicates direct interaction and dashed line indicates indirect interaction.

The ingenuity pathway analysis (IPA) tool was used to analyze the underlying network functions of the 129 genes. For clarification, IPA was used to analyze the gene regulatory networks of three refined gene signatures, respectively. The networks with direct and indirect connections are presented in Figure 6. The results indicate that the genes involved in survival prediction are mainly associated with the following biological functions: cell death and survival, cellular movement, cellular assembly and organization, cell metabolism (energy production), immune cell trafficking, and cell-to-cell signaling and interaction. Some of these genes play significant roles in cancer associated pathogeneses like hereditary disorder, connective tissue, embryonic development, and organ development disorders.

Figure 6 shows six “hub” genes,* CD44*,* ANXA2, VEGFC, CTGF, PTK2, *and* TJP1. *These “hub” genes are central to regulatory networks with no less than 10 direct/indirect connections. The details are shown in Table 2.

- CD44 gene often undergoes alternative splicing to encode different proteins in different cancer subtypes. The encoded CD44 antigen generally acts as a specific receptor for hyaluronic acid. Its functions involve ligand binding receptor, coreceptor, and organizer in cortical actin skeleton. Variations in* CD44* gene expression are reported as cell surface markers for some cancer researches [25, 26].
- *ANXA2* has been reported to be involved in cell division, proliferation, exocytosis, endocytosis, and membrane trafficking. Aberrant expression of* ANXA2* has been found in several cancers [27]. As a radiation responsive protein, ANXA2 prevents radiation-induced apoptosis by regulating nuclear factor *κB* in the nuclear translocation [28].
- The protein encoded by* VEGFC* gene is a member of the platelet-derived growth factor (PDGF)/vascular endothelial growth factor (VEGF) family. The VEGF is active in angiogenesis and endothelial cell growth and can also affect the permeability of blood vessels. High* VEGFC* expression has been implicated with nodal metastasis and poor prognosis in T1 esophageal squamous cell carcinoma [29], gastric cancer [30], gliomas [31], and breast cancer [32].
- It has been confirmed that* CTGF* gene plays an important role in cell migration, cell differentiation, and cell adhesion. CTGF protein promotes keratinocyte adhesion and migration through integrin *α*5*β*1 and activation of the FAK-MAPK signaling cascade. Aberrant* CTGF* expression is associated with many types of malignant tumors [33, 34].
- Proteins encoded by the* PTK2* gene are involved in the focal adhesions that form between cells growing in the presence of extracellular matrix constituents. Activation of this gene is an important early step in cell growth and intracellular signal transduction pathways triggered in response to certain neural peptides or to cell interactions with the extracellular matrix [35].
- *TJP1* gene encodes zonula occludens protein-1 (ZO-1), which is located on a cytoplasmic membrane surface of intercellular tight junctions. The ZO-1 has recruiting/scaffolding functions in the junctional complex of epithelial and endothelial cells [36]. It has been proven that the expression of* TJP1* is correlated with the growth and metastasis of cancer [37].

The online bioinformatics resource, DAVID v6.7 (available at http://david.abcc.ncifcrf.gov/), was also used to analyze the functions of all 129 selected genes. 120 gene ontology (GO) [38] terms and 5 Kyoto Encyclopedia of Genes and Genomes (KEGG) [39] pathways were identified. They are listed in Supplementary Tables S4 and S5. Several functions were found to be related to radiation response. They are growth factor, signal transduction, cell cycle and cell adhesion, invasion, and metastasis, and angiogenesis.

## 4. Conclusion

Accurate prediction of tumor response to radiation therapy plays a key role in personalized cancer treatment. In the present paper we have selected the genes that are significantly associated with radiation response using SAM analysis. This analysis was based on the gene expression data and SF2 values of NCI-60 cell lines. Additionally, in combination with PLS as the feature extraction method, SVM regression model was trained for SF2 prediction. Benefits derived from the gene selection, feature extraction, and nonlinear regression methods resulted in the RMSE value of only 0.011. The resulting RMSE value was much smaller than the best result (0.20) among previous studies. We also found that many genes of the selected 129-gene radiation sensitivity signature were associated with several cancer driven or cancer promoting pathways. The enriched pathways include cell signal transduction, cell adhesion, and cell binding. Additionally, an evaluation of the signature using survival analysis and gene regulatory network analysis was made. Since NCI-60 platform includes nine different cancer types, this strongly confirms the practicability of the SF2 prediction model.

## Supplementary Material

Supplementary materials contain the detailed model and parameter setting descriptions of three algorithms. Five tables and four figures are also included. These three algorithms are SAM, PLS and SVM respectively. Table S1 provides the measured and predicted SF2 values of 59 cell lines of NCI-60 platform. Table S2 provides the statistics of clinical parameters of the cancer patient datasets. Table S3 lists the symbols of 129 genes selected by SAM analysis. Table S4 and S5 provide the gene ontology terms and KEGG pathways enriched in the 129 radiation sensitivity signature genes. Figure S1 and S2 provide the survival curves of the patients in colon cancer and ovarian cancer respectively. Figure S3 and S4 provide survival analysis of colon cancer and ovarian cohorts respectively using ten hub genes reported by Eschrich et al.

## Figures and Tables

**Table 1:** Univariate Cox regression analysis for three types of cancer^a^.

Glioma (GSE4271 dataset)			Colon cancer (GSE17537 dataset)			Ovarian cancer (GSE9891 dataset)		
Gene symbol	*P* value	HR (95% CI)	Gene symbol	*P* value	HR (95% CI)	Gene symbol	*P* value	HR (95% CI)
*CKAP4 *	0.019	1.792 (1.102–2.912)	*ITGB5 *	0.042	3.017 (1.039–8.763)	*PLK2 *	0.010	1.221 (1.048–1.422)
*EPS8 *	0.003	1.785 (1.213–2.627)	*PLK2 *	0.005	1.806 (1.198–2.723)	*PLS3 *	0.032	1.243 (1.019–1.516)
*SCML1 *	<0.001	1.587 (1.235–2.040)	*CTGF *	0.039	1.427 (1.018–2.000)	*CKAP4 *	0.044	1.279 (1.007–1.626)
*RIN2 *	0.014	0.651 (0.463–0.916)	*ZNF703 *	0.020	0.566 (0.351–0.913)	*FAM129B *	0.014	0.568 (0.361–0.892)
*PPP2R3A *	0.044	0.647 (0.424–0.988)	*TM4SF1 *	0.042	2.162 (1.027–4.551)	*SRGAP1 *	0.008	1.362 (1.083–1.715)
*TJP1 *	0.007	0.520 (0.325–0.834)	*ANXA2 *	0.029	3.86 (1.147–12.996)	*RIN2 *	0.045	1.519 (1.010–2.286)
*ANKRD57 *	0.005	2.378 (1.296–4.363)	*PLEKHA1 *	0.004	5.452 (1.695–17.534)	*CTBP2 *	0.026	1.481 (1.048–2.094)
*ACTN1 *	0.047	1.179 (1.002–1.387)	*DIP2C *	0.048	2.772 (1.009–7.617)	*MYO1B *	0.001	1.463 (1.174–1.824)
*ANXA2 *	0.008	1.324 (1.075–1.631)	*LINC01137 *	0.047	0.160 (0.026–0.978)	*RAI14 *	<0.001	1.893 (1.466–2.444)
*HTRA1 *	<0.001	0.549 (0.396–0.763)	*SEPT10 *	0.017	3.564 (1.249–10.167)	*LAMB1 *	0.037	1.184 (1.010–1.388)
*TGFBI *	0.048	1.140 (1.001–1.298)	*GAS2L3 *	0.043	0.330 (0.112–0.967)	*GNA11 *	0.016	0.621 (0.422–0.914)
*DIP2C *	0.001	0.541 (0.377–0.778)	*LRMP *	0.033	1.803 (1.048–3.100)	*EMP2 *	0.031	0.770 (0.607–0.976)
*TNFRSF12A *	0.005	1.358 (1.095–1.685)	*GMFG *	0.003	3.790 (1.586–9.057)	*PFN2 *	0.023	1.316 (1.038–1.668)
*EMP2 *	0.033	1.391 (1.028–1.883)	*GIMAP6 *	0.024	1.877 (1.086–3.243)	*CD44 *	0.009	0.716 (0.557–0.921)
*CD44 *	0.049	1.238 (1.001–1.532)	*RCSD1 *	0.015	2.684 (1.213–5.939)	*RND3 *	0.006	1.366 (1.095–1.704)
*SEPT10 *	0.001	1.933 (1.294–2.886)	*CD69 *	0.044	1.361 (1.008–1.837)	*NUAK1 *	<0.001	1.303 (1.125–1.509)
*FAM114A1 *	0.020	1.384 (1.052–1.819)				*VEGFC *	0.013	1.325 (1.062–1.654)
*PRSS23 *	0.006	1.294 (1.075–1.557)				*GRIK5 *	0.014	1.515 (1.089–2.107)
*NUAK1 *	0.018	0.689 (0.506–0.939)						
*PTK2 *	0.029	0.525 (0.294–0.938)						
*ANXA2P2 *	0.008	1.497 (1.110–2.020)						
*GRIK5 *	0.014	0.719 (0.552–0.937)						
*ACVR2B *	0.006	0.622 (0.442–0.874)						
*ICAM3 *	0.013	1.305 (1.058–1.610)						
*BCOR *	0.046	1.383 (1.005–1.902)						
*IQGAP2 *	0.001	1.544 (1.182–2.018)						

^a^HR: hazard ratio; CI: confidence interval.

**Table 2:** Six hub genes identified by gene regulatory network analysis.

Gene symbol	Gene description	Location	Sequence
*CD44 *	CD44 molecule (Indian blood group)	11p13	Chromosome: 11; NC_000011.10 (35138870…35232402)
*ANXA2 *	Annexin A2	15q22.2	Chromosome: 15; NC_000015.10 (60347151…60397986, complement)
*VEGFC *	Vascular endothelial growth factor C	4q34.3	Chromosome: 4; NC_000004.12 (176683538…176792745, complement)
*CTGF *	Connective tissue growth factor	6q23.1	Chromosome: 6; NC_000006.12 (131948176…131951378, complement)
*PTK2 *	PTK2 protein tyrosine kinase 2	8q24.3	Chromosome: 8; NC_000008.11 (140658382…141001313, complement)
*TJP1 *	Tight junction protein 1 (zona occludens 1)	15q13	Chromosome: 15; NC_000015.10 (29700134…29968835, complement)
