# Supplementary material for: Nonlinear Quantitative Radiation Sensitivity Prediction Model Based on NCI-60 Cancer Cell Lines
Source: ScientificWorldJournal. 2014 Jun 17;2014:903602. doi: 10.1155/2014/903602 (PMC4083270; doi:10.1155/2014/903602)
Supplement: Supplementary file 1 — Supplementary materials contain the detailed model and parameter setting descriptions of three algorithms. Five tables and four figures are also included. These three algorithms are SAM, PLS and SVM respectively. Table S1 provides the measured and predicted SF2 values of 59 cell lines of NCI-60 platform. Table S2 provides the statistics of clinical parameters of the cancer patient datasets. Table S3 lists the symbols of 129 genes selected by SAM analysis. Table S4 and S5 provide the gene ontology terms and KEGG pathways enriched in the 129 radiation sensitivity signature genes. Figure S1 and S2 provide the survival curves of the patients in colon cancer and ovarian cancer respectively. Figure S3 and S4 provide survival analysis of colon cancer and ovarian cohorts respectively using ten hub genes reported by Eschrich et al. [file 903602.f1.pdf]

Non-linear Quantitative Radiation Sensitivity Prediction Model Based on NCI-60 Cancer Cell Lines

Chunying Zhang,[1] Luc Girard,[2,3] Amit Das,[2,4] Sun Chen,[1] Guangqiang Zheng,[1] and Kai Song[1,2,5*]

[1] *School of Chemical Engineering and Technology, Tianjin University, 300072 Tianjin, P.R. China*

[2] *Hamon Center for Therapeutic Oncology, University of Texas Southwestern Medical Center, 75390 Dallas, Texas, USA*

[3] *Department of Pharmacology, University of Texas Southwestern Medical Center, Dallas, Texas, USA, 75390*

[4] *Department of Radiation Oncology, University of Texas Southwestern Medical Center, 75390 Dallas, Texas, USA*

[5] *Department of Clinical Sciences, University of Texas Southwestern Medical Center, 75390 Dallas, Texas, USA*

*Correspondence should be addressed to Kai Song

Email: ksong@tju.edu.cn;

kai.song@utsouthwestern.edu

Tel: 1-(214)9062552

Supplementary material

Supplementary methods

Significance Analysis of Microarrays (SAM)

SAM, proposed by Tusher et al. [1], is mainly used to determine whether the changes in gene expression are statistically significant. It identifies significant genes by gene-specific t-test. SAM calculates a score d_j for gene j. In our study, d_j is used to measure the strength of the relationship between gene expression values and the radiation sensitivity. The d_j is determined as follows:

$$d_j = \frac{r_j}{s_j + s_0}, \quad j = 1, 2, ..., n \tag{S1}$$

where r_j is the linear regression coefficient of gene j, s_j is the standard error of r_j, s_0 is an exchangeability factor, n is the number of genes. The calculations of these parameters are given in [1].

SAM uses False Discovery Rate (FDR) to estimate the number of incorrectly identified significant genes. The FDR formula is:

$$\text{FDR} = \frac{Median\left(or\ 90th\ percentile\right)\ of\ the\ number\ of\ genes\ falsely\ called\ significant}{Number\ of\ genes\ called\ significant} \tag{S2}$$

In the current study, SAM gene selection was conducted using the R package “samr”. The parameter of FDR was set 1%. This corresponded to a gene score threshold of 3.67. Genes with the scores bigger than 3.67 were assumed to significantly related with radiation sensitivity and therefore were selected.

Partial Least Squares (PLS)

PLS algorithm is a useful multivariate statistical analysis tool. It attempts to model relationships between the input variables $\boldsymbol{X}_{n\times m}$ (n gene expressions, m samples) and output variables $\boldsymbol{Y}_{1\times m}$ (SF2 values). This is achieved by means of extracting a set of orthogonal Latent Variables (LVs), which are linear combinations of the original input variables. The PLS model can be described as follows [2]:

$$\boldsymbol{X}^{\mathrm{T}} = \boldsymbol{TP}^{\mathrm{T}} + \boldsymbol{E} \tag{S3}$$

$$\boldsymbol{Y}^{\mathrm{T}} = \boldsymbol{UQ}^{\mathrm{T}} + \boldsymbol{F} \tag{S4}$$

where $\boldsymbol{T}_{m\times v}$ and $\boldsymbol{U}$ are latent variable matrixes extracted from $\boldsymbol{X}$ and $\boldsymbol{Y}$. $\boldsymbol{P}$ and $\boldsymbol{Q}$ are loading matrixes, $\boldsymbol{E}$ and $\boldsymbol{F}$ are residual matrixes. Therefore, by extracting LVs, the n-dimensional original input space $\boldsymbol{X}$ is compressed into the v-dimensional LV-space. In common cases, $v<<n$, where n is the number of the original input variables and v is the number of chosen latent variables. By doing this,

PLS can effectively remove the noise and multi-collinearity of the original data, which is especially true for the gene expression data. In the current study, the derived v latent variables are used as the new input variables for the SF2 prediction model.

Support Vector Machine (SVM)

SVM is a multivariate machine learning method. In SVM regression, the key idea is to map original data space into higher or infinite dimensionality space **F** by using a nonlinear mapping [3]. This is usually conducted using "kernel function" trick. Then, in **F** space, SVM finds a linear function $f(x)$ which provides the optimum fit. The final estimated regression line (or hyperplane) is surrounded by an ε -insensitive tube with ε parameter controlling the precision of regression estimation and serving as regularization constraint to avoid model overfitting. Because of the superior performance of SVM on regression context, it has been widely-applied to the analysis of microarray data.

In this study, SVM regression was implemented using R package "e1071". The radial basis function (RBF) was used as the kernel function. There were two parameters associated with RBF kernel: C (penalty factor) and γ (RBF parameter). We utilized grid search and cross validation approach [4] to find the most suitable choices. The process was as follows:

1. Specifying parameter search ranges: $\gamma = \left[2^{-10}, 2^{3}\right]$, $C = \left[2^{-10}, 2^{7}\right]$ and dividing each of them into 100 intervals;
2. The three-fold cross-validation generalization ability of SVM with parameter pair $\left(C_i, \gamma_j\right)$ $\left(i, j = 1, 2, ..., 100\right)$ was evaluated by the following MSE:

$$\text{MSE} = \frac{1}{m_0} \sum_{k=1}^{m_0} \left(Y_k - \widehat{Y_k}\right)^2 \qquad \text{(S5)}$$

   where Y_k is the measured SF2 value of cell line k, $\widehat{Y_k}$ is the corresponding predicted SF2 value with parameter pair $\left(C_i, \gamma_j\right)$ and m_0 is the number of cell lines in the training set;
3. Selecting the pair $\left(C_i, \gamma_j\right)$ with the least MSE as the optimum parameters of final SVM model.

In this study, we obtained a best C parameter of 91.80, and a best γ parameter of 0.000977. The final prediction model was developed with the best parameters.

Supplementary Tables

Supplementary Table S1: Measured and predicted SF2 values of 59 cell lines of NCI-60 platform.[a]

Cell lines	Predicted SF2 (SVM)[b]	Predicted SF2 (LR)[c]	Measured SF2[d]	Error (SVM)	Error (LR)
BREAST_BT549	0.630	0.538	0.630	0	-0.092
BREAST_HS578T	0.785	0.562	0.790	-0.005	-0.228
BREAST_MDAMB231	0.625	0.515	0.630	-0.005	-0.115
BREAST_T47D	0.508	0.558	0.520	-0.012	0.038
CNS_U251	0.566	0.561	0.570	-0.004	-0.009
COLON_COLO205	0.683	0.524	0.690	-0.007	-0.166
COLON_HCT116	0.385	0.420	0.380	0.005	0.040
COLON_HCT15	0.396	0.382	0.400	-0.004	-0.018
COLON_HT29	0.784	0.513	0.790	-0.006	-0.277
MELAN_M14	0.415	0.620	0.420	-0.005	0.200
MELAN_MALME3M	0.802	0.620	0.800	0.002	-0.180
MELAN_SKMEL2	0.662	0.632	0.660	0.002	-0.028
MELAN_SKMEL28	0.737	0.595	0.740	-0.003	-0.145
NSCLC_A549ATCC	0.604	0.727	0.610	-0.006	0.117
NSCLC_H460	0.837	0.636	0.840	-0.003	-0.204
OVAR_OVCAR3	0.542	0.514	0.550	-0.008	-0.036
OVAR_OVCAR5	0.407	0.496	0.408	-0.001	0.088
PROSTATE_DU145	0.520	0.505	0.520	0	-0.015
PROSTATE_PC3	0.479	0.473	0.484	-0.005	-0.011
RENAL_A498	0.613	0.717	0.610	0.003	0.107
RENAL_ACHN	0.706	0.672	0.720	-0.014	-0.048
RENAL_CAKI1	0.369	0.611	0.370	-0.001	0.241
BREAST_MCF7	0.576	0.441	0.576	0	-0.135
BREAST_MDAMB435	0.195	0.494	0.179	0.015	0.314
CNS_SF539	0.820	0.661	0.820	0	-0.159
COLON_KM12	0.423	0.457	0.420	0.003	0.037
COLON_SW620	0.620	0.458	0.620	0	-0.162
LEUK_CCRFCEM	0.199	-0.072	0.185	0.014	-0.257
LEUK_HL60	0.331	0.132	0.315	0.016	-0.183
LEUK_MOLT4	0.072	-0.004	0.050	0.022	-0.054
MELAN_LOXIMVI	0.679	0.633	0.680	-0.001	-0.047
MELAN_SKMEL5	0.719	0.608	0.720	-0.001	-0.112
NSCLC_HOP62	0.169	0.667	0.164	0.005	0.503
NSCLC_NCIH23	0.102	0.508	0.086	0.016	0.422
OVAR_SKOV3	0.899	0.628	0.900	-0.001	-0.272
BREAST_MCF7ADRr	0.559	0.488	0.560	-0.001	-0.072
CNS_SF268	0.446	0.604	0.450	-0.004	0.154
CNS_SF295	0.728	0.609	0.730	-0.002	-0.121

Supplementary Table S1: Continued.[a]

Cell lines	Predicted SF2 (SVM)[b]	Predicted SF2 (LR)[c]	Measured SF2[d]	Error (SVM)	Error (LR)
CNS_SNB19	0.430	0.517	0.430	0	0.087
CNS_SNB75	0.549	0.767	0.550	-0.001	0.217
COLON_HCC-2998	0.444	0.544	0.440	0.004	0.104
LEUK_K562	0.077	0.140	0.050	0.027	0.090
LEUK_RPMI8266	0.137	0.100	0.100	0.037	0
LEUK_SR	0.118	0.437	0.070	0.048	0.367
MELAN_UACC257	0.482	0.570	0.480	0.002	0.090
MELAN_UACC62	0.522	0.591	0.520	0.002	0.071
NSCLC_EKVX	0.697	0.607	0.700	-0.003	-0.093
NSCLC_HOP92	0.431	0.607	0.430	0.001	0.177
NSCLC_NCIH226M	0.634	0.645	0.630	0.004	0.015
NSCLC_NCIH332M	0.651	0.560	0.650	0.001	-0.090
NSCLC_NCIHH522	0.430	0.348	0.430	0	-0.082
OVAR_IGROV1	0.393	0.403	0.390	0.003	0.013
OVAR_OVCAR4	0.293	0.445	0.290	0.003	0.155
OVAR_OVCAR8	0.595	0.461	0.600	-0.005	-0.139
RENAL_7860	0.666	0.639	0.660	0.006	-0.021
RENAL_RXF393	0.667	0.652	0.670	-0.003	-0.018
RENAL_SN12C	0.620	0.595	0.620	0	-0.025
RENAL_TK10	0.516	0.532	0.520	-0.004	0.012
RENAL_UO31	0.615	0.657	0.620	-0.005	0.037

[a] SF2: survival fraction at 2 Gy γ-ray radiation; LR: linear regression; SVM: support vector machine.

[b] Predicted SF2 values using SVM regression algorithm.

[c] Predicted SF2 values using LR algorithm.

[d] Measured SF2 were obtained from the study of Eschrich et al. [5].

Supplementary Table S2: Statistics of clinical parameters of the cancer patient datasets.[a]

		Glioma dataset			Colon cancer dataset			Ovarian cancer dataset	
		GSE4271[6]	GSE4412[7]		GSE17537[8]	GSE15736[8]		GSE17260[9]	GSE9891[10]
Total patients		77	85		55	177		110	278
Age[b]		45.48 ± 13.02	44.38 ± 15.47		62.31 ± 14.35	65.48 ± 13.08		NA	59.64 ± 10.58
Gender									
Female		25	53		29	81		NA	NA
Male		52	32		26	96		NA	NA
Stage									
	III	21	26	I	4	24	I		24
	IV	56	59	II	15	57	II		17
				III	19	57	III	93	214
				IV	17	39	IV	17	22
							Unknown		1
Median OS		23.75	12.97		50.20	42.27		17.00	28.50
Platform		Affymetrix	Affymetrix		Affymetrix	Affymetrix		Aglient	Affymetrix
		HG-U133A	HG-U133A		HG-U133 plus_2	HG-U133 plus_2		4×44K	HG-U133 plus_2

[a] NA: not available; OS: overall survival (months).

All cancer patient datasets can be downloaded from Gene Expression Omnibus (GEO).

[b] Age: mean ± standard deviation of patients' ages in the same dataset.

Supplementary Table S3: List of the 129 genes selected by SAM analysis.

Gene symbol						
ITGB5	*S100A16*	*CTBP2*	*YAP1*	*NQO1*	*CD53*	*IL2RG*
PLK2	*PTGR1*	*MYO1B*	*LAMB1*	*P4HA2*	*PTPRC*	*DCP2*
PARVA	*SH3BP4*	*ACTN1*	*TNFRSF12A*	*TTYH3*	*IKZF1*	*RCSD1*
BCAR3	*MYO1E*	*ANXA2*	*EPHA2*	*PRSS23*	*NSUN6*	*PRKCQ*
TUSC1	*MYOF*	*HTRA1*	*LMNA*	*GAS2L3*	*GMFG*	*SLA*
GNG12	*ZFHX3*	*TGFBI*	*GNA11*	*LRP11*	*SLAMF6*	*ICAM3*
PLS3	*TSPAN6*	*UEVLD*	*WWTR1*	*NUAK1*	*WAS*	*CD69*
CUEDC1	*FAM129B*	*LHFPL2*	*KIAA1522*	*LIF*	*PSMD5-AS1*	*BCOR*
C15orf52	*SRGAP1*	*S100A13*	*SERPINB6*	*HMOX1*	*IL23A*	*IQGAP2*
PTPN14	*ST5*	*PLEKHA1*	*IGFBP3*	*ZNF532*	*LSP1*	
CKAP4	*SCML1*	*RAI14*	*EMP2*	*CTSD*	*GRIK5*	
CTGF	*NR2F2*	*SYTL2*	*NCKAP1*	*VEGFC*	*MYO1G*	
EPS8	*RIN2*	*SH3D19*	*PFN2*	*SNX7*	*LOC285957*	
NEK6	*PPP2R3A*	*TWF1*	*CD44*	*PTK2*	*TRIM73*	
ZNF703	*TJP1*	*ARMC9*	*RND3*	*THAP10*	*RHOH*	
TM4SF1	*ATP1B1*	*CD151*	*SEPT10*	*BEAN*	*ARHGAP9*	
AGAP2-AS1	*ANKRD57*	*DIP2C*	*LMCD1*	*ANXA2P2*	*GIMAP6*	
AHNAK2	*MYO10*	*BACE1*	*FAM114A1*	*LAMB2*	*CD3D*	
CBR1	*GPX8*	*RBPMS*	*PHLDA3*	*LRMP*	*ACVR2B*	
RASAL2	*IL17RC*	*LINC01137*	*DSTN*	*CORO1A*	*RNASE6*	

Supplementary Table S4: 129 radiation sensitivity signature genes enriched gene ontology terms.

GO ID	Term	Count[a]	*P* value[b]
**Biological process (GO_TERM_BP_FAT)**			
GO:0030029	actin filament-based process	10	3.42578E-05
GO:0001568	blood vessel development	10	3.89642E-05
GO:0001944	vasculature development	10	4.70455E-05
GO:0031589	cell-substrate adhesion	7	5.02553E-05
GO:0048514	blood vessel morphogenesis	9	8.59551E-05
GO:0030036	actin cytoskeleton organization	9	1.38430E-04
GO:0045597	positive regulation of cell differentiation	9	1.51585E-04
GO:0001525	angiogenesis	7	4.80887E-04
GO:0051094	positive regulation of developmental process	9	5.59491E-04
GO:0007155	cell adhesion	14	7.82905E-04
GO:0022610	biological adhesion	14	7.93329E-04
GO:0007015	actin filament organization	5	1.39055E-03
GO:0010627	regulation of protein kinase cascade	8	1.43169E-03
GO:0042110	T cell activation	6	1.58861E-03
GO:0050870	positive regulation of T cell activation	5	1.69910E-03
GO:0007167	enzyme linked receptor protein signaling pathway	9	2.11143E-03
GO:0007010	cytoskeleton organization	10	2.61752E-03
GO:0007169	transmembrane receptor protein tyrosine kinase	7	3.98923E-03
GO:0051251	positive regulation of lymphocyte activation	5	4.12354E-03
GO:0002694	regulation of leukocyte activation	6	5.21474E-03
GO:0010740	positive regulation of protein kinase cascade	6	5.34769E-03
GO:0002696	positive regulation of leukocyte activation	5	5.64814E-03
GO:0043122	regulation of I-kappaB kinase/NF-kappaB cascade	5	5.83781E-03
GO:0050865	regulation of cell activation	6	6.49905E-03
GO:0050867	positive regulation of cell activation	5	6.63896E-03
GO:0050863	regulation of T cell activation	5	7.97237E-03
GO:0040008	regulation of growth	8	8.06074E-03
GO:0030217	T cell differentiation	4	9.52246E-03
GO:0051270	regulation of cell motion	6	9.70659E-03
GO:0001558	regulation of cell growth	6	9.91166E-03
GO:0016477	cell migration	7	1.07257E-02
GO:0046649	lymphocyte activation	6	1.09819E-02
GO:0046635	positive regulation of alpha-beta T cell activation	3	1.30971E-02
GO:0006468	protein amino acid phosphorylation	11	1.39675E-02
GO:0009967	positive regulation of signal transduction	7	1.45126E-02
GO:0006928	cell motion	9	1.45393E-02
GO:0048870	cell motility	7	1.73364E-02
GO:0051674	localization of cell	7	1.73364E-02

Supplementary Table S4: Continued.

GO ID	Term	Count[a]	*P* value[b]
GO:0051249	regulation of lymphocyte activation	5	1.76492E-02
GO:0060021	palate development	3	1.94746E-02
GO:0045582	positive regulation of T cell differentiation	3	1.94746E-02
GO:0001501	skeletal system development	7	2.05192E-02
GO:0032956	regulation of actin cytoskeleton organization	4	2.20693E-02
GO:0007160	cell-matrix adhesion	4	2.20693E-02
GO:0045621	positive regulation of lymphocyte differentiation	3	2.30604E-02
GO:0010647	positive regulation of cell communication	7	2.34591E-02
GO:0045321	leukocyte activation	6	2.35833E-02
GO:0032970	regulation of actin filament-based process	4	2.40577E-02
GO:0046634	regulation of alpha-beta T cell activation	3	2.43116E-02
GO:0001822	kidney development	4	2.68552E-02
GO:0030334	regulation of cell migration	5	2.71445E-02
GO:0043123	positive regulation of I-kappaB kinase/NF-kappaB	4	2.75807E-02
GO:0042102	positive regulation of T cell proliferation	3	2.82270E-02
GO:0006796	phosphate metabolic process	13	2.94469E-02
GO:0006793	phosphorus metabolic process	13	2.94469E-02
GO:0030098	lymphocyte differentiation	4	3.21520E-02
GO:0045892	negative regulation of transcription, DNA-dependent	7	3.27813E-02
GO:0030198	extracellular matrix organization	4	3.29501E-02
GO:0045663	positive regulation of myoblast differentiation	2	3.31893E-02
GO:0002246	healing during inflammatory response	2	3.31893E-02
GO:0000122	negative regulation of transcription from RNA	6	3.35584E-02
GO:0051253	negative regulation of RNA metabolic process	7	3.51392E-02
GO:0001655	urogenital system development	4	3.79548E-02
GO:0040012	regulation of locomotion	5	4.04879E-02
GO:0010564	regulation of cell cycle process	4	4.14951E-02
GO:0016310	phosphorylation	11	4.20875E-02
GO:0001775	cell activation	6	4.41696E-02
GO:0045580	regulation of T cell differentiation	3	4.61255E-02
GO:0045059	positive thymic T cell selection	2	4.61579E-02
GO:0031952	regulation of protein amino acid autophosphorylation	2	4.61579E-02
GO:0060284	regulation of cell development	5	4.94222E-02
**Cellular component (GO_TERM_CC_FAT)**			
GO:0015629	actin cytoskeleton	12	1.57212E-06
GO:0005886	plasma membrane	44	3.58010E-05
GO:0044459	plasma membrane part	30	1.22653E-04
GO:0005856	cytoskeleton	21	5.85483E-04
GO:0016459	myosin complex	5	9.05618E-04
GO:0016323	basolateral plasma membrane	7	2.30153E-03

Supplementary Table S4: Continued.

GO ID	Term	Count[a]	*P* value[b]
GO:0005912	adherens junction	6	3.69984E-03
GO:0005925	focal adhesion	5	4.72074E-03
GO:0005924	cell-substrate adherens junction	5	5.40917E-03
GO:0070161	anchoring junction	6	5.74083E-03
GO:0030055	cell-substrate junction	5	6.56301E-03
GO:0009986	cell surface	8	8.37752E-03
GO:0031982	vesicle	11	1.31904E-02
GO:0005604	basement membrane	4	1.50929E-02
GO:0009898	internal side of plasma membrane	7	1.86281E-02
GO:0048770	pigment granule	4	2.13910E-02
GO:0042470	melanosome	4	2.13910E-02
GO:0044421	extracellular region part	13	2.45258E-02
GO:0031410	cytoplasmic vesicle	10	2.64629E-02
GO:0031012	extracellular matrix	7	2.72444E-02
GO:0043232	intracellular non-membrane-bounded organelle	26	2.89259E-02
GO:0043228	non-membrane-bounded organelle	26	2.89259E-02
GO:0031988	membrane-bounded vesicle	9	3.48494E-02
GO:0044420	extracellular matrix part	4	4.29702E-02
GO:0005887	integral to plasma membrane	14	4.95581E-02
**Molecular function (GO_TERM_MF_FAT)**			
GO:0005546	phosphatidylinositol-4,5-bisphosphate binding	5	5.01356E-07
GO:0003779	actin binding	14	5.29497E-07
GO:0008092	cytoskeletal protein binding	16	2.66515E-06
GO:0030695	GTPase regulator activity	11	6.15459E-04
GO:0060589	nucleoside-triphosphatase regulator activity	11	7.29567E-04
GO:0035091	phosphoinositide binding	6	7.45552E-04
GO:0005543	phospholipid binding	7	1.68771E-03
GO:0019838	growth factor binding	5	6.80077E-03
GO:0005178	integrin binding	4	8.66008E-03
GO:0019899	enzyme binding	10	1.24934E-02
GO:0032403	protein complex binding	6	1.32782E-02
GO:0005520	insulin-like growth factor binding	3	1.36823E-02
GO:0005083	small GTPase regulator activity	7	1.38146E-02
GO:0003774	motor activity	5	1.89288E-02
GO:0017137	RabGTPase binding	3	1.93862E-02
GO:0003714	transcription corepressor activity	5	2.02699E-02
GO:0016564	transcription repressor activity	7	2.58504E-02
GO:0017016	RasGTPase binding	4	2.74895E-02
GO:0008047	enzyme activator activity	7	3.30755E-02
GO:0031267	small GTPase binding	4	3.58474E-02

Supplementary Table S4: Continued.

GO ID	Term	Count[a]	P value[b]
GO:0070064	proline-rich region binding	2	4.22249E-02
GO:0008289	lipid binding	8	4.24390E-02
GO:0051020	GTPase binding	4	4.33707E-02
GO:0004857	enzyme inhibitor activity	6	4.44564E-02

[a] Number of the radiation sensitivity signature genes included in each term.

[b] *P* value was derived from a modified Fisher's exact test. The smaller the *P* value was, the more significant the term was enriched.

Supplementary Table S5: 129 radiation sensitivity signature genes enriched KEGG pathways.

KEGG ID	Pathway name	*P* value[a]	Gene symbol[b]
hsa04810	Regulation of actin cytoskeleton	9.01774E-04	*PFN2, PTK2, IQGAP2, ITGB5, ACTN1, GNG12, WAS, NCKAP1*
hsa04510	Focal adhesion	3.39084E-03	*VEGFC, PTK2, LAMB2, ITGB5, ACTN1, LAMB1, PARVA*
hsa04512	ECM-receptor interaction	2.42113E-02	*LAMB2, CD44, ITGB5, LAMB1*
hsa05340	Primary immunodeficiency	2.77123E-02	*PTPRC, CD3D, IL2RG*
hsa04060	Cytokine-cytokine receptor interaction	4.36412E-02	*LIF, VEGFC, ACVR2B, IL23A, TNFRSF12A, IL2RG*

[a] *P* value was calculated in exactly the same way as GO analysis in Table S4.

[b] Radiation sensitivity signature genes included in each term.

Supplementary Figures

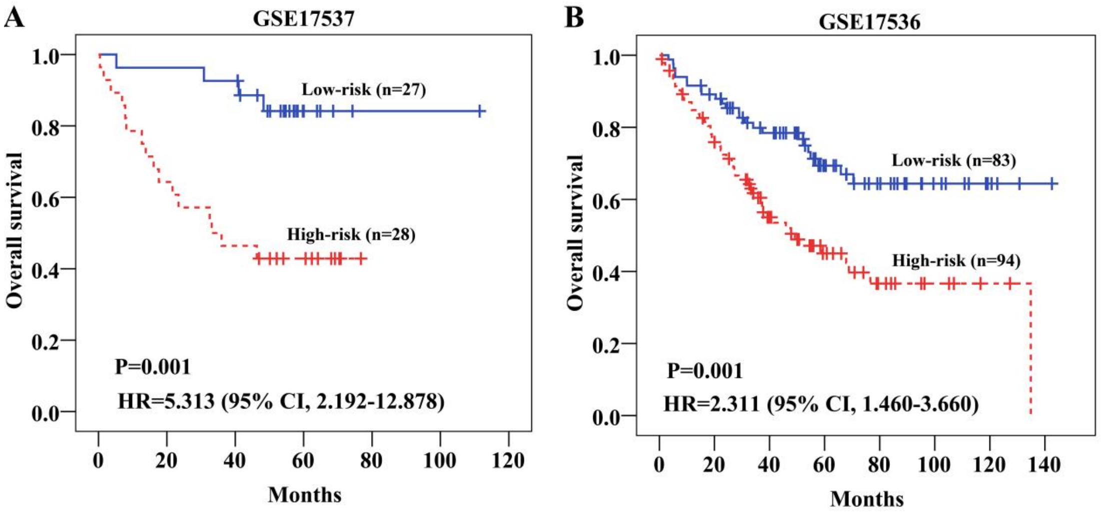

Supplementary Figure S1: Survival curves of the patients in colon cancer cohort.

HR: hazard ratio; CI: confidence interval.

The Cox regression model was trained with the 16 genes refined from the GSE17537 training dataset. The median of the estimated risk scores was used as the cutoff to divide the patients into high-risk and low-risk groups. *P* values were obtained from the log-rank test.

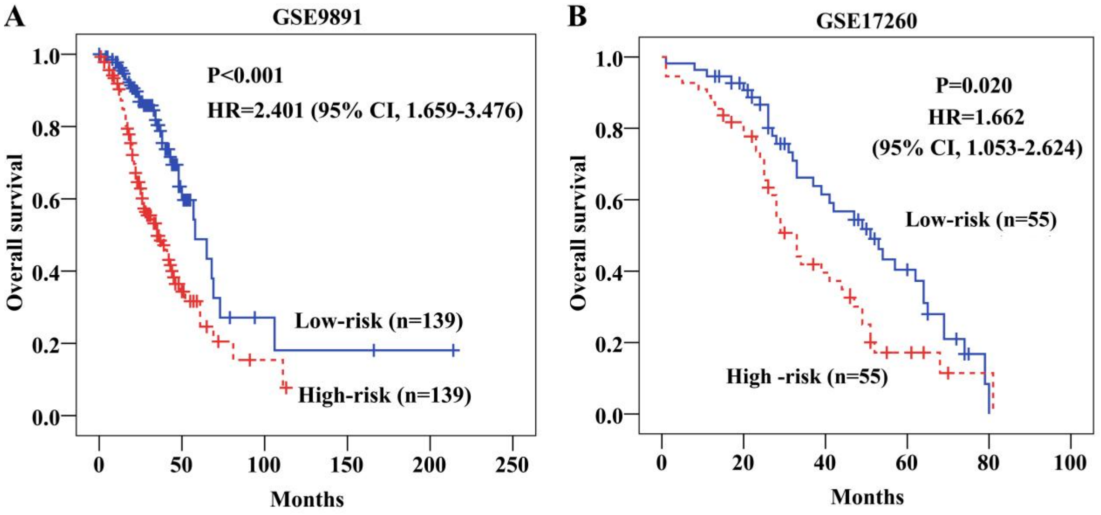

Supplementary Figure S2: Survival curves of the patients in ovarian cancer cohort.

HR: hazard ratio; CI: confidence interval

The Cox regression model was trained with the 18 genes refined from the GSE9891 training dataset. The median of the estimated risk scores was used as the cutoff to divide the patients into high-risk and low-risk groups. *P* values were obtained from the log-rank test.

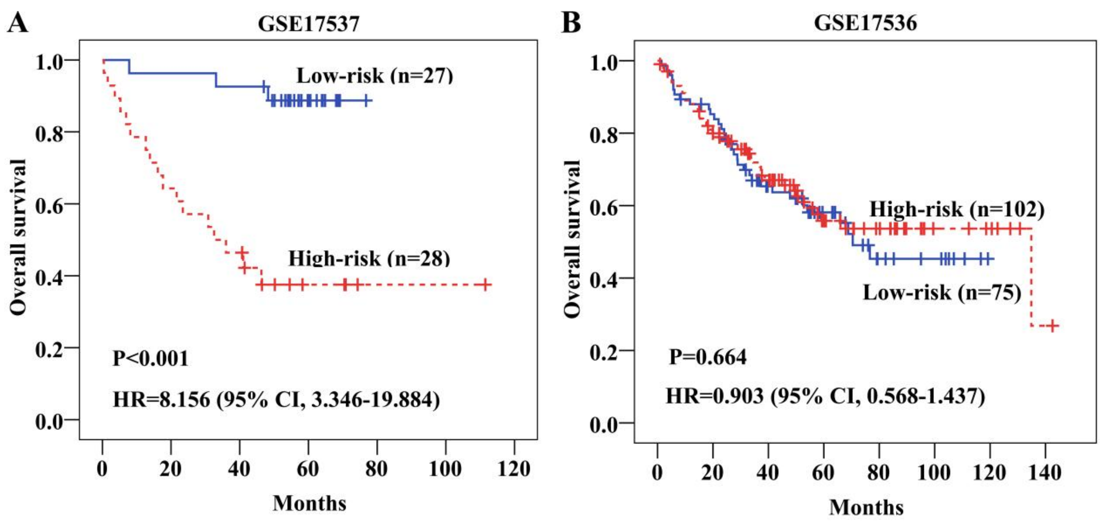

Supplementary Figure S3: Survival analysis of colon cancer cohort using ten hub genes reported by Eschrich et al.

HR: hazard ratio; CI: confidence interval.

The Cox regression model was trained on the GSE17537 training dataset. The median of the estimated risk scores was used as the cutoff to divide the patients into high-risk and low-risk groups. *P* values were obtained from the log-rank test.

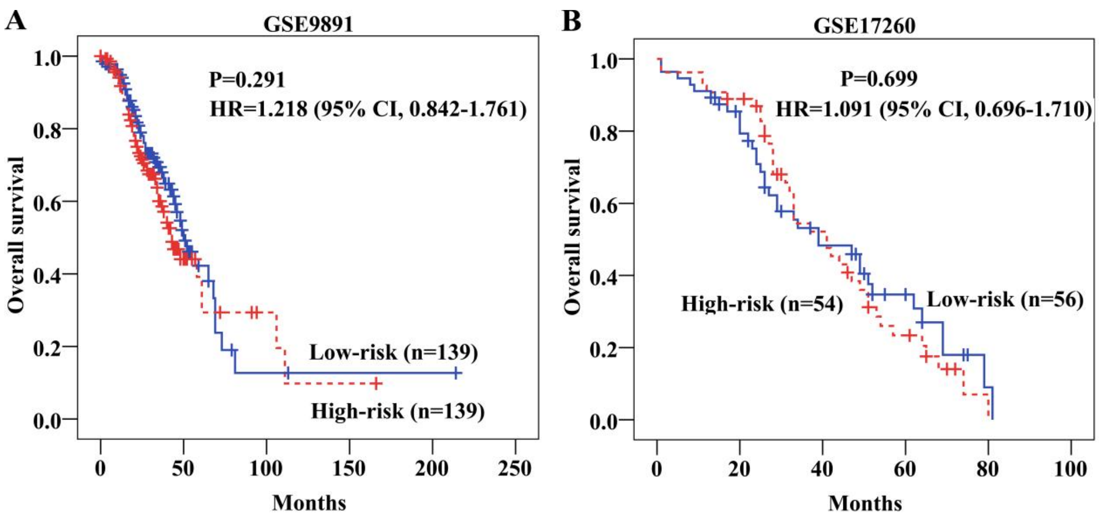

Supplementary Figure S4: Survival analysis of ovarian cancer cohort using ten hub genes reported by Eschrich et al.

HR: hazard ratio; CI: confidence interval.

The Cox regression model was trained on the GSE9891 training set. The median of the estimated risk scores was used as the cutoff to divide the patients into high-risk and low-risk groups. *P* values were obtained from the log-rank test.

Reference

[1] V. G. Tusher, R. Tibshirani, and G. Chu, "Significance analysis of microarrays applied to the ionizing radiation response," *Proceedings of the National Academy of Sciences of the United States of America*, vol. 98, no. 18, pp. 5116–5121, 2001.

[2] A.-L. Boulesteix and K. Strimmer, "Partial least squares: a versatile tool for the analysis of high-dimensional genomic data," *Briefings in Bioinformatics*, vol. 8, no. 1, pp. 32–44, 2007.

[3] A. J. Smola and B. Scholkopf, "A tutorial on support vector regression," *Statistics and Computing*, vol. 14, no. 3, pp. 199–222, 2004.

[4] A. Ben-Hur and J. Weston, "A user's guide to support vector machines," *Methods in Molecular Biology (Clifton, N.J.)*, vol. 609, pp. 223–239, 2010.

[5] S. Eschrich, H. L. Zhang, H. Y. Zhao, et al., "Systems biology modeling of the radiation sensitivity network: a biomarker discovery platform," *International Journal of Radiation Oncology* Biology* Physics*, vol. 75, no. 2, pp. 497–505, 2009.

[6] H. S. Phillips, S. Kharbanda, R. Chen, et al., "Molecular subclasses of high-grade glioma predict prognosis, delineate a pattern of disease progression, and resemble stages in neurogenesis," *Cancer Cell*, vol. 9, no. 3, pp. 157–173, 2006.

[7] W. A. Freije, F. E. Castro-Vargas, Z. Fang, et al., "Gene expression profiling of gliomas strongly predicts survival," *Cancer Research*, vol. 64, no. 18, pp. 6503–6510, 2004.

[8] J. J. Smith, N. G. Deane, F. Wu, et al., "Experimentally derived metastasis gene expression profile predicts recurrence and death in patients with colon cancer," *Gastroenterology*, vol. 138, no. 3, pp. 958–968, 2010.

[9] K. Yoshihara, A. Tajima, T. Yahata, et al., "Gene expression profile for predicting survival in advanced-stage serous ovarian cancer across two independent datasets," *PLos One*, vol. 5, no. 3, 2010.

[10] R. W. Tothill, A. V. Tinker, J. George, et al., "Novel molecular subtypes of serous and endometrioid ovarian cancer linked to clinical outcome," *Clinical Cancer Research*, vol. 14, no. 16, pp. 5198–5208, 2008.
